# Commentary: My face or yours? Event-related potential correlates of self-face processing

**DOI:** 10.3389/fpsyg.2017.00608

**Published:** 2017-04-20

**Authors:** Alejandro J. Estudillo

**Affiliations:** School of Psychology, University of Nottingham Malaysia CampusSemenyih, Malaysia

**Keywords:** own-face, P200, N170, Self-face recognition, ERP, N250

The own face is our most distinctive physical feature and the paramount representation of our own identity. In contrast to other pieces of self-related information, such as the own name, the own face is not shared with other people, and it is more strongly tied to self-awareness (e.g., Keenan et al., 2005). This makes the own face a unique piece of our physical identity and, therefore, the emblem of the self (McNeill, 1998). Behavioral, neurophysiological, and neuroimaging research have tried to unravel different aspects about the relevance of the own face (e.g., Tong and Nakayama, 1999; Brédart and Devue, 2006; Estudillo and Bindemann, 2016, 2017) and its different neural markers (Devue and Brédart, 2011).

## The N170 component

Face processing models suggest that face recognition encompasses a set of different stages, whereby the first step would imply to encode the visual stimulus as a face, the so-called structural encoding (Bruce and Young, 1986; Estudillo, 2012). The N170 is a face-specific ERP-component that has been linked to this structural encoding of the face (Eimer, 2011). Although, it has been classically assumed that this component is not affected by familiarity, Keyes et al. (2010) presented evidence showing that this component is more negative for the own face compared to familiar and unfamiliar faces. Other studies have reported similar N170 modulations by the own face (e.g., Caharel et al., 2002), but this pattern is far from being consistently found (e.g., Sui et al., 2006; Tanaka et al., 2006; Gunji et al., 2009; Pierce et al., 2011; Parketny et al., 2015). In fact, it has been suggested that the effects of the own face seen in the N170 component might be a consequence of using a small number of face stimuli (Pierce et al., 2011). The fact that the own face effect on the N170 ERP-component is not always observed casts doubts about its reliability as a valid marker of self-face processing.

## The N250 component

The N250 is another well-known face-related component that has been linked to identity activation (Schweinberger, 2011). This component is a negative deflection at occipitotemporal electrodes, which peaks ~250 ms after the presentation of a familiar face. The N250 is larger for familiar compared to unfamiliar faces. This component is also sensitive to the presentation of the own face, as it is larger for the own-face compared to unfamiliar faces (Tanaka et al., 2006). However, the N250 component does not discriminate between the processing of the own and other familiar faces as its magnitude is comparable for the own face and other familiar faces (Tanaka et al., 2006).

## The P200 component

Keyes et al. (2010) also reported an occipitotemporal P200 ERP-component, which was less positive for the own face compared to both familiar and unfamiliar faces. However, the authors did not provide an interpretation of the meaning of this component for self-face processing. Although other studies exploring self-face recognition did not report the results of the P200 component, a view of the grand mean averages shows that this component is consistently less positive for the own face compared to both familiar and unfamiliar faces (e.g., Caharel et al., 2005, 2007; Tanaka et al., 2006; Gunji et al., 2009; Parketny et al., 2015). Therefore, the cognitive meaning of the P200 for the own face is a question that needs to be unraveled as this component could be an important index of self-face processing.

The P200 component has started to attract the attention of face processing researchers (Schweinberger and Neumann, 2016). This component consists of a positive deflection which peaks between 200 and 250 ms after the presentation of a face at occipitotemporal electrodes and it is larger for less distinctive faces (Halit et al., 2000). For example, compared to own-race faces (more typical faces) other-race faces (less typical faces) elicit smaller P200 responses (Stahl et al., 2008). This component has also been interpreted as reflecting the encoding of second-order configural information. Supporting this argument, it has been shown that those faces that deviate from a prototypical average face produced a smaller P200 component (Latinus and Taylor, 2006).

The smaller P200 responses observed for the own face (Keyes et al., 2010) could reflect two different, but non-exclusive, aspects of self-face processing. On the one hand, it could reflect the distinctiveness of the own face compared to other familiar and unfamiliar faces (Tong and Nakayama, 1999; Troje and Kersten, 1999). For example, observers detect their own face faster among a set of distractors compared to other faces (Tong and Nakayama, 1999). This distinctiveness of the own face could be a consequence of overlearning, and these P200 responses could be indicative of quantitative differences due to greater exposure for the own face compared to other faces. The P200 could also index qualitative differences in terms of different processing style for the own compared to other faces, such as different recruitment of holistic (Greenberg and Goshen-Gottstein, 2009; Fuentes et al., 2013). For example, observers are slower in creating a holistic mental image of their own face than of a familiar face, but quicker in creating a mental image of a facial feature of their own face (e.g., the nose) than of a familiar face (Greenberg and Goshen-Gottstein, 2009). These results suggest that, compared to other faces, the own face is processed at a more featural level.

## Conclusions

As markers of self-face processing, the N170 and N250 components present problems of reliability and specificity. The P200 component might offer a more valid, reliable and specific index of self-face processing. However, the experimental evidence supporting this argument is scarce. A strong test for the P200 component as a valid marker of self-face processing should compare the P200 responses elicited by the own face with those elicited by other unique and distinctive facial stimuli (e.g., personally familiar faces, other-race faces, other-age faces). Smaller P200 responses for the own face compared to these other unique facial stimuli, would point the suitability of this component as an index of self-face processing.

## Author contributions

The author confirms being the sole contributor of this work and approved it for publication.

### Conflict of interest statement

The author declares that the research was conducted in the absence of any commercial or financial relationships that could be construed as a potential conflict of interest.
